# Early Surgery Reduces Psychiatric and Learning Disorder Risk in Pediatric Sleep‐Disordered Breathing

**DOI:** 10.1002/lary.70205

**Published:** 2025-10-16

**Authors:** Daniel J. Campbell, W. Jack Palmer, Leonard E. Estephan, Hani Samarah, Elliott Sina, Nicole Aaronson, William Parkes

**Affiliations:** ^1^ Department of Otolaryngology—Head and Neck Surgery Thomas Jefferson University Hospitals Philadelphia Pennsylvania USA; ^2^ Nemour's Childrens Health, Delaware Valley Wilmington Delaware USA

**Keywords:** obstructive sleep apnea, pediatrics, quality of life, sleep, surgical treatment of obstructive sleep apnea

## Abstract

**Objective:**

To determine whether the type and timing of intervention for pediatric sleep‐disordered breathing (SDB) affect the risk of developing neuropsychiatric and learning disorders.

**Methods:**

A retrospective cohort study using TriNetX, from January 2016 to March 2025, was conducted. Patients < 18 years with a diagnosis of SDB were included. Propensity score matching (1:1) based on demographics and concomitant diagnoses was performed for three comparisons: untreated versus surgical management (*n* = 152,653), untreated versus non‐surgical management (*n* = 8,634), and surgical versus non‐surgical management (*n* = 8,873). The incidence of anxiety, depressive, eating, behavioral, and learning disorders at 1 year, 5 years, and anytime post‐intervention was recorded. In surgical patients, outcomes were also stratified by timing of surgery: within versus beyond 3, 6, and 12 months of diagnosis.

**Results:**

Compared to surgery, no treatment was associated with significantly elevated risk of anxiety (risk ratio [RR] 1.44), depressive (RR 1.71), eating (RR 1.27), behavioral (RR 1.31), and learning disorders (RR 1.39) at 1 year. Compared to surgery, non‐surgical management significantly increased the risk of anxiety (RR 2.27), depressive (RR 1.69), behavioral (RR 1.24), and learning disorders (RR 2.72) at 1 year. Similar trends were observed at 5 years. Surgery within 6 months significantly reduced the risk of anxiety (RR 1.06), eating (RR 1.38), behavioral (RR 1.12), and learning disorders (RR 1.14) (*p* < 0.05 for all comparisons).

**Conclusion:**

Early surgical intervention for pediatric SDB is associated with significantly lower risks of neuropsychiatric and learning disorders compared to delayed surgery, non‐surgical management, or no treatment, necessitating validation through future prospective trials.

**Level of Evidence:**

3.

## 1 Introduction

Pediatric obstructive sleep disordered breathing (SDB) is a common, multifactorial condition affecting up to 20% of children that describes a spectrum of severity, ranging from primary snoring to obstructive sleep apnea (OSA) syndrome [1, 2]. While an estimated 1.2%–5.7% of children are affected by OSA, true prevalence is likely higher due to the need for polysomnography (PSG), symptom misattribution, and generalized lack of screening [1]. The prevalence of pediatric SDB is higher in specific subpopulations, notably in children with obesity and certain genetic syndromes or chromosomal anomalies [3, 4]. If left untreated, pediatric SDB significantly increases the risk of a wide range of health complications, including metabolic, cardiovascular, renal, pulmonary, and neuropsychiatric disorders [3]. Moreover, a variety of psychosocial consequences associated with untreated SDB are well documented, including lower academic achievement, increased school‐related behavioral disturbances, and impairments to neurocognition and attention [5]. With the rising incidence of pediatric SDB, it is increasingly important to educate healthcare providers and parents about the risks of untreated disease and systemically compare these risks to the benefits of available management options [6].

Adenotonsillectomy (AT) is the first‐line treatment for pediatric OSA in children with adenotonsillar hypertrophy according to American Academy of Pediatrics Clinical Practice Guidelines, with reported success rates of approximately 80% [7, 8]. Persistent OSA may occur in up to 29% of children following AT, even in those considered low‐risk [5]. Additional surgical management options such as lingual tonsillectomy, palatal surgery, or hypoglossal nerve stimulation have recently demonstrated success in appropriately selected patients [5]. In children for whom surgery is contraindicated or not desired, non‐invasive positive pressure ventilation (NIPPV) or the use of oral appliances have been described as alternative treatment options [7, 9]. However, non‐surgical options are often poorly tolerated, with pediatric continuous and bilevel positive airway pressure (CPAP, BIPAP) compliance rates below 50% [10]. Weight loss is beneficial for patients that are overweight or obese with pediatric SDB as well as other health‐related conditions; however, it is often a slow, inconsistent, and challenging process to maintain [7].

Despite well‐studied recognition of the neuropsychiatric and behavioral risks associated with untreated pediatric SDB [5, 11, 12, 13, 14], there exists a paucity of literature exploring whether these risks differ between non‐surgically and surgically managed cases. Existing literature comparing risks of neuropsychiatric conditions between untreated, non‐surgically managed, and surgically managed pediatric SDB is generally limited by sample size or focus on a limited number of diagnoses [15, 16, 17, 18, 19, 20]. Notably, the Childhood Adenotonsillectomy Study for Children with OSA (CHAT study) and the Pediatric Adenotonsillectomy Trial for Snoring (PATS study) identified greater improvements in behavioral, quality of life (QOL), PSG, and blood pressure measures in children managed with early AT (within 4 weeks) compared to watchful waiting [21, 22]. However, the endpoints of these clinical trials were 7 months and 12 months, respectively, and improvements in attention or executive functioning were not reported. A comprehensive assessment, which compares multiple treatment options across various long‐term endpoints, is necessary to facilitate evidence‐based discussions between healthcare providers and parents regarding management options.

Our investigation utilizes a large, multinational database to identify children diagnosed with SDB or OSA, and it intends to compare the relative risks of developing common neuropsychiatric and learning disorders between varying treatment modalities at multiple long‐term endpoints. The following disorders will be included: anxiety disorders, eating disorders, behavioral disorders, and learning disorders. Based on prior literature, we have developed the following two‐fold hypothesis: (1) surgically managed patients will have lower relative risks of neuropsychiatric and learning disorder development compared to non‐surgically managed and untreated patients, and (2) earlier surgical intervention will be associated with lower relative risks of future neuropsychiatric and learning disorder development compared to later surgical intervention.

## 2 Methods

This retrospective cohort study received an institutional board review exemption. All data were obtained and statistical analyses were performed using TriNetX. TriNetX is a global health research network that provides access to de‐identified electronic health record data of about 140 million patients from 101 healthcare organizations across 6 countries.

An initial filter of patients less than 18 years old was applied. The International Classification of Diseases 10th Revision (ICD‐10), Current Procedural Terminology (CPT), and SNOMED codes used for specific queries within our investigation are listed in Table 1. Inclusion criteria required that patients have a diagnosis of SDB or OSA. Patients were subcategorized into one of three cohorts based on intervention: (1) No management (‘Untreated’), (2) Surgical management, or (3) Non‐surgical management. Inclusion criteria further required that each patient's first instance of medical or surgical intervention must have occurred after their initial diagnosis of SDB or OSA. Surgical interventions included primary tonsillectomy and/or adenoidectomy, lingual tonsillectomy, non‐cleft uvulopalatoplasty, or hypoglossal nerve stimulation. Patients who underwent tracheostomy (CPT: 31600, 1,014,613) were excluded to isolate the effects of the above‐mentioned surgical interventions, as tracheostomy status may carry distinct psychosocial implications that could confound outcomes. Non‐surgical interventions included CPAP/BIPAP or use of an oral appliance. While intranasal steroids and leukotriene inhibitors are used for SDB, these medications are also widely prescribed for other conditions, such as allergic rhinitis or asthma, and as such were not included to avoid cohort misclassifications.

**TABLE 1:** Medical and surgical codes queried in TriNetX.

Category	Subcategory	Code (ICD‐10, CPT, SNOMED)
Sleep‐related diagnoses	OSA or SDB	G47.30, G47.33
Confounding diagnoses	Chronic tonsillitis	J35.0
	Presence of syndrome	Q90‐99
	Tonsillar hypertrophy	J35.1
	Obesity	E66, Z68.54–56
Surgical management	Tonsillectomy and/or adenoidectomy	42820, 42821, 42825, 42826, 42830, 42831, 1007181, 1007178, 1007184
	Lingual tonsillectomy	42870, 47823003
	Palatal surgery	42145, 8335001
	Hypoglossal nerve stimulation	64582, 64568
Non‐surgical management	CPAP and/or BIPAP	94660, Z99.89
	Oral appliance	E0485, E0486, 23481400
Neuropsychiatric and learning disorder diagnoses	Anxiety disorders	F41
	Depressive disorders	F32‐34, F39
	Eating disorders	F50
	Behavioral disorders	F07, F42, R46.81, F60, F90, F91.3
	Learning disorders	R48.0, F70‐73, F79, F80‐89

Abbreviations: BIPAP, bilevel positive airway pressure; CPAP, continuous positive airway pressure; OSA, obstructive sleep apnea; SDB, sleep‐disordered breathing.

One‐to‐one propensity score matching (1:1 PSM) was used to reduce the effects of confounding variables and balance baseline covariates between each group comparison. The following covariates were balanced using 1:1 PSM: age, sex, race, or diagnosis of obesity, chronic adenitis/tonsillitis, tonsillar hypertrophy, or a chromosomal syndrome. While chronic diseases of the tonsils and adenoids (ICD‐10: J35.0) and tonsillar hypertrophy (ICD‐10: J35.1) are often indications for non‐surgical or surgical management, these diagnoses were instead used as covariates to isolate the effects of SDB. The frequencies of both diagnoses varied considerably between cohorts, and chronic adenotonsillitis carries independent psychosocial risk factors and may further influence surgical timing [23].

After matching, the incidence of neuropsychiatric or learning disorders occurring in each cohort was measured. To be included, the first instance of a given neuropsychiatric or learning disorder must have occurred after the first instance of both SDB and OSA diagnosis and non‐surgical or surgical intervention. Neuropsychiatric or learning disorders were categorized into five groups: (1) anxiety disorders, (2) depressive disorders, (3) eating disorders, (4) behavioral disorders, and (5) learning disorders (Table 1). Diagnosis incidence rates were compared between each cohort at three time points following non‐surgical or surgical intervention: one year, five years, and anytime after medical or surgical intervention (“anytime”). Risk difference and risk ratio were calculated with 95% confidence intervals (CI), and a Wald test was used to calculate z‐score and *p*‐value for significance. Significance was set at *p* < 0.05.

A subanalysis was performed using only patients that received surgical management. The cohort was stratified into groupings based on the time interval between their first recorded SDB diagnosis and surgical intervention. Three time intervals were included: 3 months, 6 months, and 1 year. This allowed for the construction of three separate analyses based on time from initial SDB diagnosis to surgical intervention—surgery performed before versus after: (1) 3 months after initial SDB diagnosis, (2) 6 months after initial SDB diagnosis, or (3) 1 year after initial SDB diagnosis. The same statistical analysis was then used to compare the risk difference in neuropsychiatric and learning diagnosis incidence based on latency to surgery.

## 3 Results

Prior to 1:1 PSM, there were over 312,000 patients in the untreated cohort, over 212,000 patients in the surgical management cohort, and over 8900 patients in the non‐surgical management cohort. Unmatched total patient count differed slightly between analyses due to continuous data refresh and query processing variability inherent to TriNetX. After 1:1 PSM, inter‐cohort covariate variability was significantly reduced, and final cohort sizes were 152,653 patients (untreated vs. surgical management), 8,634 patients (untreated vs. non‐surgical management), and 8,873 patients (surgical vs. non‐surgical management).

Incidence, risk ratio (RR), and the resultant *p*‐value for each cohort comparison at varying time constraints post‐intervention are listed in Tables 2, 3, 4. The risk of neuropsychiatric and learning disorders differed significantly within each cohort comparison. Compared to surgically managed disease, the untreated cohort had an increased risk of every neuropsychiatric and learning disorder grouping at one year, five years, and anytime after surgical intervention (*p* < 0.0001 for all except for eating disorders; *p* < 0.05 for eating disorders) (Table 2).

**TABLE 2:** Risk ratio for development of neuropsychiatric and learning disorders at varying time intervals: Untreated versus surgically managed SDB.

	1 year			5 years			Anytime		
	Incidence	RR	*p*	Incidence	RR	*p*	Incidence	RR	*p*
Anxiety disorder		1.44 (1.34, 1.55)	< 0.0001		1.21 (1.16, 1.25)	< 0.0001		1.17 (1.14, 1.21)	< 0.0001
Untreated	1874 (1.212)			6329 (4.09)			8959 (6.07)		
Surgical	1.313 (0.841)			5298 (3.39)			7723 (5.19)		
Depressive disorder		1.71 (1.52, 1.92)	< 0.0001		1.30 (1.23, 1.37)	< 0.0001		1.23 (1.18, 1.28)	< 0.0001
Untreated	765 (0.484)			2802 (1.77)			4842 (3.22)		
Surgical	449 (0.283)			2172 (1.37)			3971 (2.63)		
Eating disorder		1.27 (1.09, 1.49)	0.0035		1.13 (1.03, 1.24)	0.0137		1.23 (1.13, 1.34)	< 0.0001
Untreated	343 (0.216)			904 (0.569)			1139 (0.75)		
Surgical	271 (0.170)			803 (0.505)			926 (0.61)		
Behavioral disorder		1.31 (1.23, 1.39)	< 0.0001		1.09 (1.05, 1.13)	< 0.0001		1.12 (1.08, 1.15)	< 0.0001
Untreated	2236 (1.458)			7050 (4.60)			8570 (5.85)		
Surgical	1730 (1.115)			6541 (4.23)			7774 (5.25)		
Learning disorder		1.39 (1.33, 1.45)	< 0.0001		1.26 (1.22, 1.30)	< 0.0001		1.24 (1.20, 1.27)	< 0.0001
Untreated	4884 (3.851)			9536 (7.52)			10,107 (8.34)		
Surgical	3519 (2.774)			7581 (5.98)			8161 (6.75)		

*Note*: Incidence column is notated as incidence (% total in respective cohort). Risk ratio is reported as ±95% confidence interval.

Abbreviation: RR, risk ratio.

**TABLE 3:** Risk ratio for development of neuropsychiatric and learning disorders at varying time intervals: Untreated versus non‐surgically managed SDB.

	1 year			5 years			Anytime		
	Incidence	RR	*p*	Incidence	RR	*p*	Incidence	RR	*p*
Anxiety disorder		0.727 (0.59, 0.89)	0.0022		0.782 (0.69, 0.89)	0.0001		0.808 (0.72, 0.90)	0.0002
Untreated	156 (1.99)			403 (5.13)			511 (6.51)		
Non‐surgical	210 (2.73)			504 (6.56)			619 (8.06)		
Depressive disorder		1.01 (0.76, 1.33)	0.9727		0.898 (0.76, 1.07)	0.218		1.01 (0.87, 1.17)	0.9274
Untreated	98 (1.20)			242 (2.95)			324 (3.95)		
Non‐surgical	97 (1.19)			268 (3.29)			320 (3.93)		
Eating disorder		1.03 (0.63, 1.67)	0.9143		0.962 (0.72, 1.29)	0.7985		0.923 (0.71, 1.21)	0.5599
Untreated	33 (0.387)			86 (1.01)			102 (1.20)		
Non‐surgical	32 (0.377)			89 (1.05)			110 (1.30)		
Behavioral disorder		1.20 (0.93, 1.53)	0.1556		1.32 (1.13, 1.54)	0.0005		1.30 (1.14, 1.50)	0.0002
Untreated	135 (1.69)			360 (4.51)			445 (5.58)		
Non‐surgical	113 (1.41)			274 (3.43)			342 (4.28)		
Learning disorder		0.530 (0.47, 0.61)	< 0.0001		0.64 (0.59, 0.71)	< 0.0001		0.665 (0.61, 0.73)	< 0.0001
Untreated	361 (5.63)			728 (11.35)			811 (12.6)		
Non‐surgical	467 (10.62)			777 (17.67)			836 (19.0)		

*Note*: Incidence column is notated as incidence (% total in respective cohort). Risk ratio is reported as ±95% confidence interval.

Abbreviation: RR, risk ratio.

**TABLE 4:** Risk ratio for development of neuropsychiatric and learning disorders at varying time intervals: Surgically managed versus non‐surgically managed SDB.

	1 year			5 years			Anytime		
	Incidence	RR	*p*	Incidence	RR	*p*	Incidence	RR	*p*
Anxiety disorder		2.271 (1.80, 2.87)	< 0.0001		1.731 (1.51, 1.98)	< 0.0001		1.532 (1.36, 1.72)	< 0.0001
Non‐surgical	218 (2.77)			525 (6.66)			645 (8.19)		
Surgical	101 (1.22)			319 (3.85)			443 (5.34)		
Depressive disorder		1.688 (1.23, 2.32)	0.0010		1.534 (1.28, 1.84)	< 0.0001		1.322 (1.13, 1.55)	0.0006
Non‐surgical	101 (1.21)			280 (3.34)			336 (4.01)		
Surgical	61 (0.714)			186 (2.18)			259 (3.03)		
Eating disorder		1.58 (0.92, 2.73)	0.0979		1.483 (1.07, 2.05)	0.0162		1.543 (1.15, 2.07)	0.0035
Non‐surgical	33 (0.378)			90 (1.03)			112 (1.28)		
Surgical	21 (0.239)			61 (0.70)			73 (0.832)		
Behavioral disorder		1.238 (0.95, 1.62)	0.1150		1.024 (0.87, 1.20)	0.7760		1.012 (0.877, 1.168)	0.8680
Non‐surgical	119 (1.45)			284 (3.47)			358 (4.37)		
Surgical	97 (1.17)			280 (3.39)			357 (4.32)		
Learning disorder		2.722 (2.35, 3.16)	< 0.0001		2.024 (1.83, 2.24)	< 0.0001		1.938 (1.76, 2.13)	< 0.0001
Non‐surgical	478 (10.51)			799 (17.56)			859 (18.8)		
Surgical	250 (3.86)			8.68 (8.68)			631 (9.74)		

*Note*: Incidence column is notated as incidence (% total in respective cohort). Risk ratio is reported as ±95% confidence interval.

Abbreviation: RR, risk ratio.

Table 3 displays the outcomes between the untreated cohort and the non‐surgically managed cohort. There were no significant differences in the risk of depressive or eating disorders between the two cohorts. Compared to non‐surgically managed patients, there was a higher risk of behavioral disorders in untreated patients at 5 years (RR 1.32; 95% CI, 1.13–1.54; *p* = 0.0005) and at the “anytime” constraint (RR 1.30; 95% CI 1.14–1.50; *p* = 0.0002) post intervention. However, there was a higher risk of anxiety (*p* < 0.01) and learning disorders (*p* < 0.0001) in the non‐surgically managed group across all time points.

Table 4 displays the outcomes between the non‐surgical management cohort and surgical management cohort. The non‐surgically managed cohort had a significantly higher risk of anxiety (*p* < 0.0001), depressive disorders (*p* ≤ 0.01), and learning disorders (*p* < 0.0001) across all time points. Additionally, the risk of developing an eating disorder was higher in the non‐surgically managed group at 5 years (RR 1.48; 95% CI, 1.07–2.05; *p* = 0.0162) and anytime (RR 1.543; 95% CI, 1.15–2.07; *p* = 0.0035); this difference was not observed at the 1‐year time point. There were no significant differences observed across anytime point for behavioral disorders.

Table 5 displays the outcomes for the subanalysis of only surgically‐managed patients. This compared the risks of developing neuropsychiatric or learning disorders based on the time latency between initial SDB diagnosis and surgery. Delaying surgery beyond 1 year was associated with a significantly higher risk in all neuropsychiatric and behavioral disorders (*p* ≤ 0.0001 for all comparisons except for depressive disorders; *p* < 0.05 for depressive disorders). With the exception of depression (RR 1.011; 95% CI, 0.95–1.08; *p* = 0.752), delaying surgery beyond 6 months was also associated with a significantly higher risk in all other neuropsychiatric and behavioral disorders (*p* < 0.05). At the diagnosis‐to‐surgery window of 3 months, there was a higher risk of delaying surgery for eating disorders (RR 1.33; 95% CI 1.19–1.49; *p* < 0.0001), behavioral disorders (RR 1.04; 95% CI 1.00–1.08; 0.041), and learning disorders (RR 1.097; 95% CI, 1.06–1.14; *p* < 0.0001). However, there was no significant risk increase for anxiety disorders, and there was a lower risk for development of depressive disorders (RR 0.907; 95% CI, 0.86–0.956; *p* = 0.0003).

**TABLE 5:** Risk ratio for development of neuropsychiatric and learning disorders based on time latency between initial diagnosis and surgical intervention.

Window from diagnosis to surgery	3 months			6 months			1 year		
	Incidence	RR	*p*	Incidence	RR	*p*	Incidence	RR	*p*
Anxiety disorder		1.00 (0.964, 1.038)	0.9973		1.059 (1.01, 1.11)	0.0153		1.171 (1.10, 1.24)	< 0.0001
After timepoint	5197 (6.00)			3435 (6.42)			2136 (7.13)		
Before timepoint	5258 (6.00)			3294 (6.06)			1857 (6.09)		
Depressive disorder		0.907 (0.86, 0.956)	0.0003		1.011 (0.95, 1.08)	0.7521		1.094 (1.01, 1.19)	0.0369
After timepoint	2534 (2.85)			1734 (3.14)			1097 (3.53)		
Before timepoint	2804 (3.14)			1724 (3.11)			1008 (3.23)		
Eating disorder		1.333 (1.19, 1.49)	< 0.0001		1.376 (1.21, 1.57)	< 0.0001		1.396 (1.18, 1.66)	0.0001
After timepoint	739 (0.829)			536 (0.967)			313 (1.00)		
Before timepoint	557 (0.622)			392 (0.703)			226 (0.72)		
Behavioral disorder		1.04 (1.00, 1.08)	0.0408		1.124 (1.07, 1.18)	< 0.0001		1.205 (1.13, 1.28)	< 0.0001
After timepoint	5212 (6.07)			3362 (6.35)			1974 (6.68)		
Before timepoint	5106 (5.84)			3057 (5.65)			1680 (5.55)		
Learning disorder		1.097 (1.06, 1.14)	< 0.0001		1.136 (1.08, 1.19)	< 0.0001		1.231 (1.15, 1.32)	< 0.0001
After timepoint	4546 (6.85)			2826 (7.34)			1486 (7.39)		
Before timepoint	4622 (6.24)			2880 (6.46)			1471 (6.00)		

*Note*: Major columns (3 months, 6 months, 1 year) represent differing timepoints between initial diagnosis of SDB and surgical intervention. Each row represents the risk of developing a given neuropsychiatric or learning disorder based on surgery being performed before or after a given timepoint. Incidence column is notated as incidence (% total in respective cohort). Risk ratio is reported as ±95% confidence interval.

Abbreviations: RR, risk ratio; SDB, sleep disordered breathing.

## 4 Discussion

This study demonstrates that surgical management of pediatric SDB is associated with a lower risk of neuropsychiatric and learning disorders compared to both untreated and non‐surgically managed patients. Furthermore, early surgical intervention, particularly within six months of diagnosis, yields superior outcomes to delayed intervention. Leveraging a cohort of over 613,000 pediatric patients with SDB, this analysis utilized 1:1 PSM to minimize variability in head‐to‐head comparisons. The study included 28 unique neuropsychiatric and learning disorder diagnosis codes, grouped into five distinct categories, to assess long‐term, multi‐year outcomes. These findings provide a comprehensive contribution to pediatric SDB literature and may help guide informed discussions between healthcare providers and parents.

The first aim of this study was to assess which treatment modality—surgical, non‐surgical, or no intervention—yields the lowest relative risk of neuropsychiatric and learning disorders at various post‐treatment intervals. Consistent with our hypothesis, surgical management of pediatric SDB was associated with superior outcomes compared to non‐surgical or untreated patients, a trend observed at 1 year, 5 years, and anytime following intervention. These findings underscore existing data and national guidelines recommending AT (i.e., surgical management) as first‐line treatment [7].

A detailed analysis of our first aim reveals that learning disorders exhibited the strongest association with treatment modality. Compared to surgical management, non‐surgical management was associated with a significantly higher RR of developing learning disorders: 2.722 (95% CI, 2.35–3.16) at 1 year and 2.024 (95% CI, 1.83–2.24) at 5 years (*p* < 0.0001). A similar trend was observed in comparison against the untreated cohort, with RRs of 1.39 (95% CI, 1.33–1.45) at 1 year and 1.26 (95% CI, 1.22–1.30) at 5 years (*p* < 0.0001). Depressive and anxiety disorders demonstrated the second strongest signal, favoring surgical management compared to both non‐surgical management and untreated disease. Specifically, compared to surgical intervention, non‐surgically managed disease was associated with a RR of 2.271 (95% CI, 1.80–2.87) for anxiety (*p* < 0.0001) and 1.688 (95% CI, 1.23–2.32) for depression (*p* = 0.001) at 1 year post‐intervention, while untreated disease was associated with RRs of 1.44 (95% CI, 1.34–1.55) for anxiety and 1.71 (95% CI, 1.52–1.92) for depression (both *p* < 0.0001) at the same time interval.

While surgical intervention appears to portend a favorable risk ratio for disease development compared to non‐surgical and no intervention, it is noted that the risk factors generally decline as the time from surgery increases. For example, compared to surgical intervention, untreated patients had a depressive disorder RR of 1.71 (95% CI, 1.52–1.92) at 1 year, 1.30 (95% CI, 1.23–1.37) at 5 years, and 1.23 (95% CI, 1.18–1.28) at anytime after surgery. The declining risk ratios observed likely reflect the natural accumulation of neuropsychiatric and learning disorders over time. While surgery may provide an early and measurable risk reduction according to our findings, there likely remains an inherent population‐level risk for these disorders in all patients. As follow‐up time lengthens, the cumulative incidence of diagnoses rises, narrowing the relative difference. Additionally, it is known that persistent OSA after surgery may occur in over 25% of children [24]. Thus, it can be inferred that a subset of surgical patients likely experienced residual or recurrent OSA, a signal that TriNetX is unfortunately unable to identify due to its inherent database limitations. As OSA is a known risk factor for neuropsychiatric disease development, this unrecognized signal of persistent OSA may further attenuate the long‐term benefit.

The present data underscore a recent clinical trial suggesting that children with SDB managed with surgery have lower healthcare utilization, as these children likely experienced fewer visits associated with neuropsychiatric or learning disorders [25]. However, these findings contrast somewhat with the CHAT clinical trial, which did not observe significant improvements in attention or executive function following surgical treatment as assessed by neuropsychological testing [22]. Differences in both sample size and length of follow‐up may have contributed to this discordance. The CHAT trial evaluated 464 children with a 7‐month endpoint, whereas our study analyzed several hundred thousand patients with multiyear endpoints. Nonetheless, these results highlight the need for larger, longer‐term, randomized clinical trials to further investigate these associations.

The second clinical aim focused exclusively on pediatric SDB patients who underwent surgical management, examining whether the timing of surgery from initial diagnosis influenced the risk of developing neuropsychiatric or learning disorders. While the CHAT and PATS trials defined “early AT” as surgery within four weeks of randomization, our study adopted more practical timeframes of 3 months, 6 months, and 1 year to reflect real‐world clinical practice scheduling constraints [26, 27]. Our findings indicate that early AT, defined as surgery within six months of diagnosis, was consistently associated with a reduced risk of all assessed neuropsychiatric and learning disorders with the exception of depressive disorders. Relative risk values ranged from 1.059 (95% CI, 1.01–1.11; *p* = 0.0153) for anxiety disorders to 1.376 (95% CI, 1.21–1.57; *p* < 0.0001) for eating disorders. Moreover, when using a one‐year cutoff for early AT, a lower risk of depression with earlier surgery was also observed (RR 1.171; 95% CI, 1.10–1.24; *p* < 0.0001).

Interestingly, a recent large‐scale cohort study by Xiao et al. using the Swedish Patient Register suggested that AT was associated with higher future risks of stress‐related psychiatric disorders [19]. While their study design did employ population‐ and sibling‐matched cohorts, due to database limitations, it did not require patients to carry diagnosis codes for OSA or SDB, nor did it control for other clinical patient attributes. In contrast, our study required all patients to have OSA or SDB diagnosis codes and employed PSM to balance possible confounding clinical attributes. Our investigation does contradict Xiao et al.'s overarching conclusion by suggesting that surgical intervention is associated with a lower risk of acquiring neuropsychiatric diagnoses compared to non‐surgical interventions. Additionally, our secondary analysis of surgical timing adds the possibility that delayed surgery may be a contributing factor at play as well, a signal that perhaps could have been driving Xiao et al.'s conclusion but could not be identified without the appropriate diagnosis codes to compute time from diagnosis to surgery.

Non‐surgical management of pediatric SDB did not consistently reduce the risk of neuropsychiatric and learning disorders compared to untreated disease. While it was associated with a lower risk of behavioral disorders at 5 years (RR 1.32; 95% CI, 1.13–1.54; *p* = 0.0005), no significant differences in the development of depressive or eating disorders were observed. Notably, non‐surgical management actually increased the risk of anxiety and learning disorders at all time points (*p* < 0.01) compared to untreated SDB. This finding may be attributable to a form of cohort selection bias inherent to the TriNetX platform. TriNetX does not provide stratification by OSA severity (i.e., mild, moderate, severe). It may be inferred that patients prescribed PAP therapy had more severe disease. Thus, our findings may underscore previous literature that demonstrates an association between OSA disease burden and neuropsychiatric disease development [3, 28]. Additionally, the untreated group may also include patients with limited access to healthcare, making them less likely to either receive treatment or a subsequent psychiatric diagnosis.

As in any study, it is vital to consider both the statistical and clinical significance of reported results. While absolute differences in risk appear small—for example, the risk of developing an anxiety disorder at 1 year was 2.77% in the non‐surgically managed group compared to 1.22% in the surgical group—the overall trend favoring surgical management across nearly all neuropsychiatric and learning disorders remains clinically relevant. Even modest risk ratios, such as 1.1 or 1.2, translate to a 10%–20% higher relative likelihood of developing a condition based on treatment modality. From a clinical and parental perspective, these cumulative risks are meaningful and may influence treatment decisions in children.

This study has several limitations. While our 1:1 PSM controlled for several factors that could influence the likelihood of surgery, additional unaccounted confounders may still exist. TriNetX does not provide access to granular PSG data, preventing matching based on AHI and precluding stratification by OSA severity. Future studies with access to OSA severity and patient‐specific exam findings may seek to assess patients who underwent surgery but later required NIPPV. Medical therapies such as intranasal steroids or leukotriene inhibitors were excluded due to nonspecific use across other conditions, which could obscure cohort accuracy. Additionally, the poorer outcomes associated with non‐surgical management may, in part, reflect poor adherence to PAP therapy. However, adherence data downloads from PAP devices are not accessible through TriNetX. Moreover, while our query included 28 unique ICD‐10, CPT, and SNOMED codes for neuropsychiatric and learning disorders—many of which were broad umbrella codes themselves—some diagnostic codes may have been missed, potentially limiting overall disease capture. Furthermore, the TriNetX platform does not distinguish provider type in the initial SDB diagnosis. Thus, it is plausible that if an otolaryngologist made the initial diagnosis, time to surgery may be shorter or symptoms may have existed prior to the initial SDB chart documentation. Also, while our study controlled for age via PSM, it is plausible that age at the time of surgery itself may influence neuropsychiatric diagnosis development—a research variable that may be addressed in future investigations. Finally, the retrospective nature of this study presents inherent limitations that may be improved with future prospective and randomized study designs.

## 5 Conclusion

Early surgical intervention for pediatric sleep‐disordered breathing is associated with significantly lower risks of neuropsychiatric and learning disorders compared to delayed surgery, non‐surgical management, or no treatment. Future prospective clinical trials are needed to further validate these associations and refine management strategies.

## Conflicts of Interest

The authors declare no conflicts of interest.

## Data Availability

The data that support the findings of this study are available from TriNetX. Restrictions apply to the availability of these data, which were used under license for this study. Data are available from http://trinetx.com with the permission of TriNetX.
